# Use of a Robotic Sampler (PIPER) for Evaluation of Particulate Matter Exposure and Eczema in Preschoolers

**DOI:** 10.3390/ijerph13020242

**Published:** 2016-02-19

**Authors:** Lokesh Shah, Gediminas Mainelis, Maya Ramagopal, Kathleen Black, Stuart L. Shalat

**Affiliations:** 1 Rutgers-Robert Wood Johnson Medical School, New Brunswick, NJ 08901, USA; lokesh006@gmail.com; 2 Department of Environmental Sciences, Rutgers The State University of New Jersey, New Brunswick, NJ 08901, USA; mainelis@envsci.rutgers.edu; 3 Department of Pediatrics, Rutgers-Robert Wood Johnson Medical School, New Brunswick, NJ 08903, USA; ramagoma@rwjms.rutgers.edu; 4 Environmental and Occupational Health Sciences Institute, Piscataway, NJ 08854, USA; kgblack@eohsi.rutgers.edu; 5 Division of Environmental Health, School of Public Health, Georgia State University, Atlanta, GA 30303, USA

**Keywords:** eczema, asthma, children, robot, particulate matter

## Abstract

While the association of eczema with asthma is well recognized, little research has focused on the potential role of inhalable exposures and eczema. While indoor air quality is important in the development of respiratory disease as children in the U.S. spend the majority of their time indoors, relatively little research has focused on correlated non-respiratory conditions. This study examined the relationship between particulate matter (PM) exposures in preschool age children and major correlates of asthma, such as wheeze and eczema. Air sampling was carried out using a robotic (PIPER) child-sampling surrogate. This study enrolled 128 participants, 57 male and 71 female children. Ages ranged from 3 to 58 months with the mean age of 29.3 months. A comparison of subjects with and without eczema showed a difference in the natural log (ln) of PM collected from the PIPER air sampling (*p* = 0.049). PIPER’s sampling observed an association between the ln PM concentrations and eczema, but not an association with wheezing history in pre-school children. Our findings are consistent with the hypothesis of the role of the microenvironment in mediating atopic dermatitis, which is one of the predictors of persistent asthma. Our findings also support the use of PIPER in its ability to model and sample the microenvironment of young children.

## 1. Introduction

While indoor air quality is known to play a significant role in the development of respiratory disorders, far less is known about its potential role in childhood skin conditions, such as eczema. Given that children in the U.S. spend the majority of their time indoors [1] it would seem logical to explore exposures in the indoor environment. Studies have shown that sensitization to indoor allergens is a risk factor for the development of asthma [2]. Like asthma, eczema may be exacerbated by antigens found in the environment such as dust, pollen, and animal dander. In addition to their role in triggering allergic responses, antigens may also be involved in the disease etiology.

The link between eczema and respiratory disorders such as childhood wheeze and asthma has been the subject of extensive research. The result is there is little doubt that atopy is a major factor in the etiologies of these two very different categories of conditions [3,4,5,6]. Asthma is an example of a Th2-mediated hypersensitivity reaction, which has long been known to have an etiology influenced by the environment [7]. Atopic dermatitis in young children, which is a predictor of persistent asthma later in life; is believed to be mediated by similar hypersensitivity mechanisms triggered by an environmental allergen [8]. A proper understanding of these etiologies, therefore, requires a detailed study of the microenvironment containing these allergens. While the role of inhalable particulate matter (PM) is obvious in respiratory conditions, the potential mechanism is less obvious for dermatologic conditions.

The current methods for evaluating young children’s exposure to airborne particulate matter (PM) rely on stationary area air sampling. The limitation with this method of PM collection is that it does not take into account re-suspension of PM following activity. In understanding the inhaled microenvironment, the concept of the “personal dust cloud” was developed, which accounts for particles generated from personal activities and local sources [9,10]. Yet while this is particularly important in young children’s exposure because of the amount of time they spend playing on the floor, it is also impractical to measure with personal samplers. A mobile, robotic surrogate, the Pretoddler Inhalable Environmental Robot (PIPER) as described by Shalat *et al.* attempts to collect more relevant data on children’s exposure by simulating the near floor microenvironment. PIPER is an autonomous robot that has been shown to improve the ability to estimate concentrations of resuspendable PM in a young child’s breathing zone [11]. PIPER is programmed to act as a surrogate for child behavior given its ability to re-suspend particulate matter as well as collect air samples at various heights from the floor [11,12].

Quantitatively determining the PM in a child’s environment during the period before asthma can be diagnosed may improve our understanding the origins of this disease. The Asthma Predictive Index is a tool that has been developed to determine the likelihood of persistent asthma in children. In the Asthma Predictive Index for preschool children, the occurrence of eczema is associated with recurrent wheeze developing into clinically diagnosed asthma later in childhood [13]. What is currently unknown is the relationship between the air microenvironment and the risk factors associated with the development of persistent asthma, notably wheezing history and eczema. Perhaps of greater importance is a fact that while the symptoms of childhood asthma may remit over time, the forced expiratory volumes remain persistently decreased even in the absence of symptoms in a majority of patients suggesting another persistent trigger [14]. Understanding the microenvironment may improve our understanding of the relationship between the initial phenotypes and the consequential respiratory pathology. In this study, air samples were measured in the homes of children up to 5 years of age with and without asthma using PIPER. In addition to the PM measurements, a basic questionnaire based upon the International Study of Asthma and Allergies in Children (ISAAC) questionnaire was administered, which included questions regarding wheezing episodes and whether or not they were diagnosed with eczema [15]. The aim of the overall study was to determine what relationship, if any, exists between the PM exposures in preschool age children with major symptoms and comorbidities of asthma, specifically wheezing and eczema. The goal of this paper is to explore whether or not there was a significant difference in the PM concentrations between the aforementioned variables, with particular focus on the relationship between inhalation exposure to PM and early childhood eczema.

## 2. Experimental Section

In this study, we recruited parents, who had at least one child between 3 and 60 months of age, to allow us to take air measurements in their home (Table 1) and to complete with the assistance of a trained technician questionnaires on the child’s play activities and health.

Participation was voluntary, and participants were recruited from two pediatric clinics in New Jersey and through a local health fair. Those who agreed to participate signed informed consent approved by the Rutgers University-IRB (IRB Protocols 0220070004 and 0220110115). One set of 78 homes was obtained from the first PIPER Assessment study completed in 2011 (IRB Protocol 0220070004) and an additional 50 residences were obtained from a second related study completed in 2013 (IRB Protocol 0220110115). Air sampling was performed in the room the parents indicated was the child’s main play area, based upon a brief activity questionnaire. At the time of scheduling home visits, participants were asked to follow their normal cleaning routine. Information was collected on type of residence, heating and cooling system, floor surface type, temperature and humidity and time of sampling. A questionnaire, which was completed by the parents, asked for the exposure to cigarette smoking, the child’s respiratory health including wheezing history, as well as, a history of diagnosis of eczema. In addition to the PM sampling, one mold spore sampler and two additional samplers to collect for endotoxin and allergens were deployed in the same room. Participating homes had at least one child who resided in the home between the ages of 3 months and 5 years. In the initial 75 homes, both a stationary sampler and PIPER were deployed for sampling; in the subsequent 50 homes only PIPER was employed. After setup the room was not sampled for the first 30 min in order to allow for settling of dust disturbed in the setup process. Air sampling was subsequently carried out for a minimum of 90 min in each residence. Both sets of PM samples were collected employing the same protocol and details of the sampling have been previously reported [11,16].

The PM concentrations were obtained from air sampling measurements using the Pre-toddler Inhalable Particulate Environmental Robotic sampler (PIPER). In summary, PIPER is a four-wheeled robot with a variable height air sampling tower upon which air sampling heads are mounted [11]. PM was collected with a Button Aerosol Sampler (SKC Inc., Eighty Four, PA, USA), which collects inhalable particles <100 μm in mean aerodynamic diameter. The Button Aerosol Sampler was operated at a flow rate of 10 L/min provided by a Leland Legacy pump (SKC Inc., Eighty Four, PA, USA).

### Use of a Sampling Surrogate Robot

The Pre-toddler Inhalable Particulate Environmental Robotic sampler (PIPER) is a mobile air sampler platform that was designed to act a surrogate for estimating exposure for young children (less than 3 years of age). This is necessary since conventional sampling equipment is both too heavy and cumbersome for young children to wear. PIPER was developed to meet the need for an air sampler that takes into consideration Ozkaynak’s “personal dust cloud” phenomenon [9]. The personal dust cloud reflects the resuspension of PM from activities and the local environment. PIPER is programmed with activity profiles, which are based upon prior video recordings of children at play in their own homes, to create a personal dust cloud representative of a child. It has been shown that PIPER detects, on average inhalable PM, at a level 2–3 times higher than stationary platforms [11], it has also been used to link children’s exposure to early childhood asthma, wheeze, and eczema [17]. This suggests that PIPER is detecting aspects of the inhalable particulate environment of a young child’s microenvironment not afforded by other tools. PIPER has the additional benefit of being reproducible in sampling resuspension of PM in the environment, by eliminating the day-to-day variation that an individual child’s play activity may introduce in sampling the household environment on a single day.

The goal of this study was to determine if any relationship existed between the PM concentrations measured through PIPER and any of the phenotypes of hypersensitivity reactions—notably, wheezing and eczema. Wheezing history was further divided into wheezing the past year and wheezing ever in the subject’s lifetime. The frequency of those who had wheezed in the past year and wheezed ever was found to be different by Sign test.

When the air sampling measurements were conducted, a questionnaire as previously mentioned was administered to the parents. The questionnaire asked health related questions about the child and characterized household demographics and potential exposures. The pertinent variables studied in this paper include: whether the participant had ever wheezed; whether the participant had ever wheezed specifically in the past year; whether the participant has eczema; the gender of the participant; and the age of the participant in months.

Statistical analyses were performed at α = 0.05 level using PASW Statistics 18 (SPSS Inc., Chicago, IL, USA), and STATA 14.0 (Stata Corp, College Station, TX, USA) using data from the two studies mentioned above, *n* = 128. The distribution of PM concentrations was not normally distributed and so the ladder of power computation (*gladder* function in STATA), which provides graphic evaluation of distribution, was employed to determine the best transformation for normalizing the data. In order to statistically confirm using “wheeze ever” *vs.* “wheezing in the past year” as separate variables, a nonparametric sign test was performed. ANOVA was employed to evaluate the difference in (normalized) PM exposures between individuals with and without eczema. Finally, separate multivariate logistic regression models were computed with presence or absence of “wheeze ever”, “wheezing in the past year”, and eczema as the dependent variable and age (months), gender and natural log of the PM concentration as the independent variables to evaluate the association of PM exposure and eczema.

## 3. Results and Discussion

The study enrolled 128 participants, 57 male and 71 female children. While not by study design, only two households had resident cigarette smokers. Detailed results of the sampling for endotoxin, mold and allergens have been previously reported [10,16]. As described in the method section, nonparametric Sign tests were performed to determine if there was a significant difference in the frequency of those who reported “wheezing ever” and “wheezing in the past year.” Seventy-four reported wheezing ever, and 68 reported wheezing during the past year. The Sign test was statistically significant with *p* = 0.031. This discrepancy may be due to unmeasured variables such as the use of recent inhaled corticosteroid therapy. Given this finding, “wheezing in the past year” was considered as a separate variable from “wheezing ever” in subsequent analyses.

The ages of subjects ranged from 3 to 58 months with the mean age of 29.3 months with 95% of the subjects between 29.2 months and 31.4 months. There was no statistically significant difference of the mean ages between genders (male mean age of 30.2 months, female mean age of 28.2 months, *p* = 0.353).

Further analysis was performed to determine if there were differences in wheezing history and diagnosis of eczema between genders. Twenty-one percent of the males had eczema, 59% had wheezed in the past year, and 66% had ever wheezed in their lifetimes. Among females, 25% had eczema, 35% had wheezed in the past year, and 42% had ever wheezed (Table 2). These findings are in agreement with national and statewide rates for New Jersey reflecting a greater prevalence of asthma in boys than in girls [18].

The levels of PM exposure, after being normalized with a log base e transformation, by reported symptoms are presented in Table 3.

Based upon analysis (gladder) of the shape and agreement of various normalizing transformations for PM exposure the natural log (ln) transformation was selected. In order to make the results easier to interpret in terms of exposure, the anti-log of the determined value is also provided. As seen in Table 3 the subjects with eczema had a mean potential PM exposure of 68.71 µg/m^3^, (*n* = 29) and those without eczema a mean of 50.91 µg/m^3^ (*n* = 99). When those with and without eczema were compared in an ANOVA analysis the two groups were just statistically significantly different (*p* = 0.0490).

In the multivariate logistic regression modeling, neither of the wheeze variables was statistically significantly associated with ln PM (wheeze ever; OR = 1.19, *p* = 0.51, 95% CI 0.71–1.98; wheeze year; OR = 1.08, *p* = 0.79, 95% CI 0.65–1.79). For both wheeze variables gender was a strong and statistically significant predicator in the model with males over twice as likely to have reported symptoms (*p* < 0.007), while age was not (*p* > 0.50). However, the results of the multivariate logistic regression analysis for eczema, observed a stronger and approached a statistically significant association OR = 1.81 with *p* = 0.052, and 95% CI (0.996, 3.301). In that same analysis, neither age in months (OR = 0.99, 95% CI 0.95–1.02) or gender (OR = 0.87, 95% CI 0.37–2.02) were statistically associated with eczema. In addition when the top quartile of ln PM (mid-point 84.83 µg/m^3^) exposures were compared to all other subjects they were statistically significantly more likely to have a reported history of eczema (OR = 2.85; *p* = 0.022, 95% CI 1.16–6.97).

Our findings lend support to the notion that atopic dermatitis and asthma can share an air quality mediated hypersensitivity reaction. For example, a Korean study of kindergarten-aged children using stationary air samplers showed an improvement of eczema severity after reducing indoor air pollutants [19]. Many studies posit that eczema is a skin disorder manifestation of a systemic immunologic reaction [20]. The pathogenesis of asthma and eczema share the Th2 cell hyperactivity; importantly, the Th2 cell over-activity is preceded by a hyper-reactivity to environmental triggers, either cutaneous or airborne.

PM concentration (transformed), however, was not associated with the history of wheezing. On average, there was no association with higher levels of measured PM and having ever wheezed. This finding is not completely unexpected as wheezing is common secondary to viral illnesses in this age group. The underlying pathology is also fundamentally different in that inhaled corticosteroids do not improve clinical outcomes in children from viral-induced wheezing as they would in asthma-related wheezing [21]. Wheezing is often an unreliable indicator of asthma in young children [22]. Our findings suggest that PM concentration cannot explain the wheezing phenotype.

Our findings are also consistent with observations of school-age children and adolescents regarding gender differences, which show that males typically have more wheezing episodes than females [23]. From our sample, we observed more male children had reported histories of “wheeze ever” than female children. Our study, however, demonstrates the gender difference pattern at earlier ages by studying the preschool population.

There are several limitations of the current study. One is that for this study family history data was not collected for the entire study population. A family history of first-degree relatives with asthma is a factor in predicting the persistence of asthma. Our data could be further analyzed contrasting our sample between those with a family history and those without a family history of asthma. A further limitation would be the absence of detailed clinical information on the age of first diagnosis and severity of the child’s eczema and asthma. This would perhaps further contribute to a better appreciation of the chronology of these conditions in relation to environmental factors. Additionally, while in the initial study population no detectable allergens were observed in air samples of the first 25 subjects, it is certainly possible that allergens may and likely are an important factor in the makeup of the resuspendable fraction of housedust. The study is also cross-sectional, but the indoor environment, particularly with regard to our focus on resuspendable PM, tends to be more stable over time and across seasons in the residences considered in this study [10].

## 4. Conclusions

Although the findings are only suggestive, our findings are consistent with the hypothesis that presence of PM in the personal dust cloud of young children is relevant to the development of eczema. It is therefore important to explore the exact nature and composition of housedust in order to identify if constituents beyond what is already known with regard to allergens may play a role in the early manifestations of this condition. Our future goal is to utilize and optimize PIPER to elucidate the role of the microenvironment in hypersensitivity reactions, notably, eczema and asthma.

## Figures and Tables

**Table 1:** Frequency of age distribution and history of eczema.

Age Distribution	*n*	Eczema	No Eczema
3 ≤ 12 months	13	3	10
12 ≤ 24 months	16	3	13
24 ≤ 36 months	71	19	52
36 ≤ 48 months	17	4	13
48 ≤ 60 months	11	0	11

Abbreviation: *n*, sample size.

**Table 2:** Demographics of subjects and history of wheeze and eczema.

Demographics	*n*	Mean Age ± SD Age (mo)	% Wheezed in the Past Year	% Wheezed Ever in Lifetime	% with Eczema
Males	57	30.2 ± 10.9	59%	66%	21%
Females	71	28.2 ± 12.9	35%	42%	25%

Abbreviations: *n*, sample size; SD, standard deviation.

**Table 3:** Concentrations (direct and natural log transformed) of Particulate Matter (PM) Exposure by Symptoms.

Characteristic	*n*	Mean ln(PM)	SD ln(PM)	Mean PM (µg/m^3^)	Median PM (µg/m^3^)	Range of PM (µg/m^3^)
Wheezing in Past Year	62	4.01	0.73	55.15	54.60	(6.62–291.52)
No Wheezing in Past Year	66	3.99	0.71	54.05	47.00	(13.41–378.56)
Wheezing Ever in Lifetime	57	4.03	0.77	56.26	56.26	(6.62–338.66)
No Wheezing Ever in Lifetime	71	3.96	0.64	52.54	47.00	(13.41–378.56)
Eczema	29	4.23	0.89	68.71	56.26	(8.19–378.56)
No Eczema	99	3.93	0.65	50.91	48.91	(6.62–272.14)

Abbreviations: *n*, sample size; PM, particulate matter; SD, standard deviation.

## References

[1] Klepeis N.E., Nelson W.C., Ott W.R., Robinson J.P., Tsang A.M., Switzer P., Behar J.V., Hern S.C., Engelmann W.H. (2001). The National Human Activity Pattern Survey (NHAPS): A resource for assessing exposure to environmental pollutants. J. Expo. Anal. Environ. Epidemiol..

[2] Custovic A., Simpson A., Woodcock A. (1998). Importance of indoor allergens in the induction of allergy and elicitation of allergic disease. Allergy.

[3] Selgrade M.K., Lemanske R.F., Gilmour M.I., Neas L.M., Ward M.D., Henneberger P.K., Weissman D.N., Hoppin J.A., Dietert R.R., Sly P.D. (2006). Induction of asthma and the environment: What we know and need to know. Environ. Health Perspect..

[4] Fu T., Lemanske R.F., Gilmour M.I., Neas L.M., Ward M.D., Henneberger P.K., Weissman D.N., Hoppin J.A., Dietert R.R., Sly P.D. (2014). Eczema and sensitization to common allergens in the United States: A multiehtnic, population-based study. Pediatr. Dermatol..

[5] Cohn L., Elias J.A., Chupp G.L. (2005). Asthma: Mechanisms of disease persistence and progression. Annu. Rev. Immunol..

[6] Kiley J., Smith R., Noel P. (2007). Asthma phenotypes. Curr. Opin. Pulm. Med..

[7] Walter M.J., Holtzman M.J. (2005). A centennial history of research on asthma pathogenesis. Am. J. Respir. Cell. Mol. Biol..

[8] Rudikoff D., Lebwohl M. (1998). Atopic dermatitis. Lancet.

[9] Ozkaynak H., Xue J., Spengler J., Wallace L., Pellizzari E., Jenkins P. (1996). Personal exposure to airborne particles and metals: Results from the Particle TEAM Study in Riverside, California. J. Expo. Anal. Environ. Epidemiol..

[10] Allen R., Larson T., Sheppard L., Wallace L., Liu L.J. (2003). Use of real-time light scattering data to estimate the contribution of infiltrated and indoor-generated particles to indoor air. Environ. Sci. Technol..

[11] Shalat S.L., Stambler A.A., Wang Z., Mainelis G., Emoekpere O.H., Hernandez M., Lioy P.J., Black K. (2011). Development and in-home testing of the Pretoddler Inhalable Particulate Environmental Robotic (PIPER Mk IV) sampler. Environ. Sci. Technol..

[12] Wang Z., Shalat S.L., Black K., Lioy P.J., Stambler A.A., Emoekpere O.H., Hernandez M., Han T., Ramagopal M., Mainelis G. (2012). Use of a robotic sampling platform to assess young children's exposure to indoor bioaerosols. Indoor Air.

[13] Castro-Rodriguez J.A., Holberg C.J., Wright A.L., Martinez F.D. (2000). A clinical index to define risk of asthma in young children with recurrent wheezing. Am. J. Respir. Crit. Care Med..

[14] Vonk J.M., Postma D.S., Boezen H.M., Grol M.H., Schouten J.P., Koëter G.H., Gerritsen J. (2004). Childhood factors associated with asthma remission after 30 years follow up. Thorax.

[15] Asher M.I., Keil U., Anderson H.R., Beasley R., Crane J., Martibez F.D. (1995). International Study of Asthma and Allergies in Childhood (ISAAC): Rationale and methods. Eur. Respir. J..

[16] Shalat S.L., Lioy P.J., Schmeelck K., Mainelis G. (2007). Improving estimation of indoor exposure to inhalable particles for children in the first year of life. J. Air. Waste. Manag. Assoc..

[17] Ramagopal M., Wang Z., Black K., Hernandez M., Stambler A.A., Emoekpere O.H., Mainelis G., Shalat S.L. (2014). Improved exposure characterization with robotic (PIPER) sampling and association with children's respiratory symptoms, asthma and eczema. J. Expo. Anal. Environ. Epidemiol..

[18] Winer R.A., Qin X., Harrington T., Moorman J., Zahran H. (2012). Asthma incidence among children and adults: Findings from the behavioral risk factor surveillance system asthma call-back survey—United States, 2006–2008. J. Asthma.

[19] Kim H.O., Kim J.H., Cho S.I., Chung B.Y., Ahn I.S., Lee C.H., Park C.W. (2013). Improvement of atopic dermatitis severity after reducing indoor air pollutants. Ann. Dermatol..

[20] Leung D.Y., Bieber T. (2003). Atopic dermatitis. Lancet.

[21] Fernandes R.M., Bialy L.M., Vandermeer B., Tjosvold L., Plint A.C., Patel H., Johnson D.W., Klassen T.P., Hartling L. (2013). Glucocorticoids for acute viral bronchiolitis in infants and young children. Cochrane Database Syst. Rev..

[22] Martinez F.D., Wright A.L., Taussig L.M., Holberg C.J., Halonen M., Morgan W.J. (1995). Asthma and wheezing in the first six years of life. The Group Health Medical Associates. N. Engl. J. Med..

[23] Strachan D.P., Butland B.K., Anderson H.R. (1996). Incidence and prognosis of asthma and wheezing illness from early childhood to age 33 in a national British cohort. BMJ.

